# A short-term intervention with selenium affects expression of genes implicated in the epithelial-to-mesenchymal transition in the prostate

**DOI:** 10.18632/oncotarget.14551

**Published:** 2017-01-06

**Authors:** Dieuwertje E.G. Kok, Lambertus A.L.M. Kiemeney, Gerald W. Verhaegh, Jack A. Schalken, Emile N.J.T. van Lin, J.P. Michiel Sedelaar, J. Alfred Witjes, Christina A. Hulsbergen - van de Kaa, Pieter van't Veer, Ellen Kampman, Lydia A. Afman

**Affiliations:** ^1^ Division of Human Nutrition, Wageningen University, Wageningen, The Netherlands; ^2^ Department for Health Evidence, Radboud University Medical Center, Nijmegen, The Netherlands; ^3^ Department of Urology, Radboud university Medical Center, Nijmegen, The Netherlands; ^4^ Strahlentherapie Bonn Rhein Sieg, Wesel, Germany; ^5^ Department of Pathology, Radboud University Medical Center, Nijmegen, The Netherlands

**Keywords:** selenium, prostatic neoplasms, gene expression, microarray, EMT

## Abstract

In parallel with the inconsistency in observational studies and chemoprevention trials, the mechanisms by which selenium affects prostate cancer risk have not been elucidated. We conducted a randomized, placebo-controlled trial to examine the effects of a short-term intervention with selenium on gene expression in non-malignant prostate tissue. Twenty-three men received 300 μg selenium per day in the form of selenized yeast (n=12) or a placebo (n=11) during 5 weeks. Prostate biopsies collected from the transition zone before and after intervention were analysed for 15 participants (n=8 selenium, n=7 placebo). Pathway analyses revealed that the intervention with selenium was associated with down-regulated expression of genes involved in cellular migration, invasion, remodeling and immune responses. Specifically, expression of well-established epithelial markers, such as E-cadherin and epithelial cell adhesion molecule *EPCAM*, was up-regulated, while the mesenchymal markers vimentin and fibronectin were down-regulated after intervention with selenium. This implies an inhibitory effect of selenium on the epithelial-to-mesenchymal transition (EMT). Moreover, selenium was associated with down-regulated expression of genes involved in wound healing and inflammation; processes which are both related to EMT. In conclusion, our explorative data showed that selenium affected expression of genes implicated in EMT in the transition zone of the prostate.

## INTRODUCTION

Based on early observational and intervention studies, it has been suggested that an adequate status or intake of selenium may protect against prostate cancer [1–4]. The Nutritional Prevention of Cancer (NPC) trial showed that 200 μg selenized yeast per day reduced the incidence of prostate cancer, and advanced prostate cancer in particular [2]. More recent studies, however, did not consistently confirm a protective effect of selenium for prostate cancer [5–8]. Results of the Selenium and Vitamin E Cancer Prevention (SELECT) trial demonstrated that supplements with 200 μg *L*-selenomethionine did not decrease the incidence of prostate cancer among men in the general population [6, 9]. For men with high toenail selenium levels, a slightly increased risk of high-grade prostate cancer was found after supplementation with selenium [7]. Similarly, in men at high risk for prostate cancer, 200-400 μg of selenized yeast per day was not effective for prostate cancer prevention [8].

The relatively high baseline selenium levels of the participants are often considered a plausible explanation for the unanticipated findings of subsequent trials as compared to the early NPC trial [6, 10, 11]. Intake and status of selenium in most European countries are relatively low as compared to the USA [10, 12]. This supports further exploration of the role of selenium for prostate cancer prevention in these populations with marginal selenium status [11]. Rather than initiating another large intervention trial, we advocate to first gain further insight into the mechanisms underlying the role of selenium in cancer biology and other health related outcomes.

In parallel with the inconsistency in observational studies and chemoprevention trials, the mechanisms by which selenium may affect prostate cancer risk have not been elucidated. Supplementation with selenium resulted in increased levels of selenium in the prostate as shown in previous studies [13]. Furthermore, it was demonstrated that after supplementation with 200 μg *L*-selenomethionine per day differences exist in gene expression profiles in human prostate tissue as compared to a placebo group [14]. Whether these differences in gene expression underlie possible chemopreventive properties is not clear, because *L*-selenomethionine itself did not show preventive effects for prostate cancer in the SELECT trial [6]. Other forms of selenium, such as selenized yeast, have been shown to be effective for the prevention of prostate cancer in a subgroup of participants with a low baseline selenium status [1, 2], however, detailed information on the molecular effects in prostate tissue is lacking.

The aim of the current study was to obtain more insight into the molecular pathways affected by selenium in the prostate. Therefore, we examined whole-genome expression profiles in non-malignant prostate tissue before and after a 5-week intervention with selenized yeast in a randomized, placebo-controlled trial in The Netherlands. The collection of prostate tissue before and after intervention allowed us to compare changes in gene expression within individuals over time.

## RESULTS

From June 2007 to October 2010, 281 men were assessed for their eligibility to participate in this study. Of these, 23 men were finally enrolled; 12 participants were randomized to the selenium group and 11 participants to the placebo group (Supplementary Figure 1). Baseline characteristics of the participants are presented in Table 1. The median duration of the intervention period was 35 days (interquartile range (IQR): 31; 35). Compliance, as assessed by pill count and inspection of research diaries, ranged from 94% to 100%.

**Table 1:** Characteristics of the participants at baseline and after the intervention with selenium or placebo

	All	Participants who completed the intervention		Participants who were eligible for microarray analysis	
		Placebo	Selenium	Placebo	Selenium
**Sociodemographic**					
Number of participants	23	11	12	7	8
Age at start intervention (years)	67.5 (65.0-72.3)	69.5 (63.0-72.6)	67.1 (65.2-71.2)	65.6 (61.9-73.1)	68.4 (65.2-71.9)
Body mass index (kg/m^2^)	26.2 (24.2-28.1)	26.2 (24.7-28.5)	26.4 (23.8-28.0)	26.2 (24.7-28.5)	25.5 (23.5-27.8)
Smoking					
Current	4 (17%)	1 (9%)	3 (25%)	-	1 (13%)
Former	13 (57%)	6 (55%)	7 (67%)	5 (71%)	5 (63%)
Never	6 (26%)	4 (36%)	2 (8%)	2 (29%)	2 (25%)
Use of dietary supplements (current)	7 (30%)	4 (36%)	3 (25%)	2 (29%)	2 (25%)
**Clinical**					
Prediagnostic PSA levels (ng/mL)	8.0 (4.5-10.3)	7.7 (3.8-11.0)	9.4 (6.0-10.2)	4.0 (3.0-11.0)	9.6 (6.4-10.2)
Diagnosis					
No evidence of malignancy	1 (4%)	-	1 (8%)	-	1 (13%)
HGPIN	5 (22%)	4 (36%)	1 (8%)	3 (43%)	1 (13%)
Prostate cancer	17 (74%)	7 (64%)	10 (83%)	4 (57%)	6 (75%)
Gleason score at biopsy^a^					
<7	10 (59%)	3 (43%)	7 (70%)	3 (75%)	5 (83%)
≥7	7 (41%)	4 (57%)	3 (30%)	1 (25%)	1 (17%)
Chronic inflammation at baseline^b^					
No inflammation	8 (35%)	3 (27%)	5 (42%)	2 (29%)	2 (25%)
<10% of the study biopsy	13 (57%)	7 (64%)	6 (50%)	5 (71%)	5 (63%)
10-50% of the study biopsy	1 (4%)	-	1 (8%)	-	1 (13%)
Unknown	1 (4%)	1 (9%)	-	-	-
Type of treatment / clinical follow-up					
Re-biopsy	9 (39%)	4 (36%)	5 (42%)	3 (43%)	4 (50%)
Radical prostatectomy	6 (26%)	4 (36%)	2 (17%)	2 (29%)	-
Radiotherapy	8 (35%)	3 (27%)	5 (42%)	2 (29%)	4 (50%)
**Intervention**					
Duration of intervention period (days)	35 (31-35)	35 (34-35)	33 (28-35)	35 (34-35)	35 (32-35)
Time between collection of prostate tissue (days)	64 (35-98)	65 (36-98)	64 (33-96)	65 (36-112)	49 (32-96)
**Selenium levels**					
Toenail selenium (mg/kg)	at baseline	0.45 (0.37-0.50)	0.43 (0.37-0.48)	0.44 (0.37-0.48)	0.42 (0.37-0.60)
Serum selenium levels (μmol/L)	at baseline	1.06 (0.92-1.18)	1.00 (0.92-1.08)^c^	1.09 (1.04-1.18)	1.06 (0.93-1.17)
	after intervention	1.11 (0.95-1.25)^d^	2.36 (1.74-2.98)^c,e^	1.12 (0.98-1.23)	2.82 (2.30-3.04)^f^

^a^Only for participants with prostate cancer,

^b^Chronic inflammation in the prostate biopsies was defined as infiltration of mononuclear cells at the periglandular stroma. The extent of inflammation was classified as affected volume (%) of the study biopsy.

^c^One participant from the selenium group was excluded, because data on serum selenium levels at baseline and after intervention were not available,

^d^One participant in the placebo group was excluded, because sample collection after the intervention failed.

^e^Statistically significant if compared to baseline levels (p=0.004, Wilcoxon Signed Rank test).

^f^Statistically significant if compared to baseline levels (p=0.012, Wilcoxon Signed Rank test).

Data presented as median (interquartile range) or numbers (%). Statistical tests were only performed for toenail and serum selenium levels.

Abbreviations: *HGPIN* high-grade prostatic intraepithelial neoplasia, *PSA* prostate specific antigen.

### Levels of selenium

Baseline levels of serum (p=0.562) or toenail (p=0.449) selenium did not differ between the two groups (Table 1). As compared to baseline values, the serum selenium levels after intervention increased in the selenium group (median increase 1.44 μmol/L, IQR 0.66; 1.92, p=0.004), but not in the placebo group (median increase 0.02 μmol/L, IQR -0.04; 0.18, p=0.314).

### Microarray analyses

Good quality RNA from prostate biopsies from the transition zone without evidence of malignancy was available before and after the intervention for 15 participants, resulting in a total of 30 microarrays used in this study (Supplementary Figure 1). After Robust Multichip Average (RMA) normalization and filtering, 18,437 genes were considered expressed and were included in further analyses (Supplementary Figure 2). Comparisons between the selenium and placebo group showed that for 2765 genes pairwise expression changes (after intervention minus before intervention) differed (Limma p-value <0.05). Subsequent within-group comparisons of individual gene expression profiles before and after intervention revealed that of these 2765 genes, expression of 915 genes (524 down-regulated and 391 up-regulated) was changed in the selenium group. In the placebo group expression changes were observed for 1380 out of 2765 genes, of which 661 were down-regulated and 719 up-regulated (Supplementary Table 1). At baseline, differences in gene expression exist between the selenium and placebo group (n=2624 genes with Limma p-value <0.05).

### Biological pathway analyses

**Figure 1:**
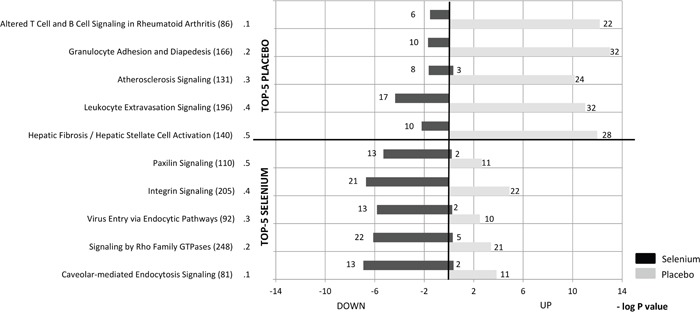
The top-5 pathways, identified by IPA Canonical Pathway Analysis, which are most significantly regulated by the intervention with selenium (lower panel) or placebo (upper panel) The numbers behind the pathways indicate the number of genes that belong to that pathway and the numbers behind the bars represent the number of differentially expressed genes within that pathway (these genes had a p-value of <0.05 in the within- and between-group comparisons). Significance of the pathways, as assessed by the Fisher’s Exact test, is expressed by a (-log2) p-value.

To obtain more insight into the role of the differentially expressed genes, pathway analyses were conducted. The top-5 regulated canonical pathways are presented for both the selenium and the placebo group in Figure 1. The intervention with selenium was associated with down-regulated expression of genes involved in signaling pathways related to inflammation, cellular immune response and cellular growth, proliferation and development. Opposite effects were observed in the placebo group with expression of genes involved in pathways related to cellular immune responses being up-regulated.

**Figure 2:**
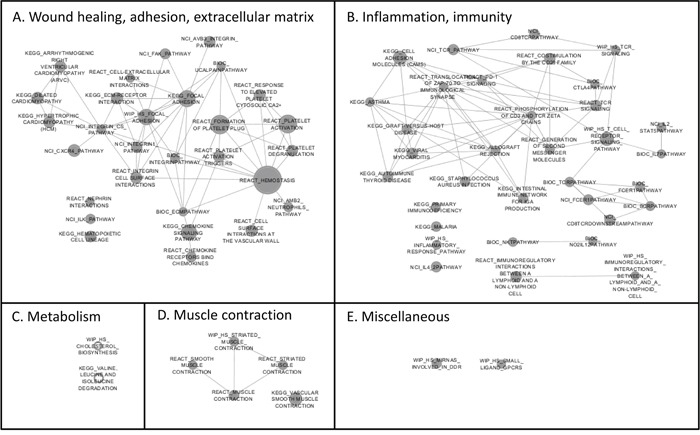
Enrichment map of the Gene Set Enrichment Analysis (GSEA) reflecting the gene sets that are enriched after the intervention with selenium The gene sets are presented according to clusters which were assigned a label based on their common function or annotation. The clusters are: **A**. wound healing, cellular adhesion and extracellular matrix interactions, **B**. inflammation and immunity, **C**. metabolism, **D**. muscle contraction, **E**. miscellaneous. Down-regulated gene sets are visualized as solid grey nodes, while up-regulated gene sets are white nodes with a grey border. Size of the nodes represents the number of genes involved. If there are overlapping genes between gene sets, the gene sets are connected through a line.

A gene set enrichment analysis (GSEA) was performed to explore the consistency of our findings for the selenium supplementation. Gene sets enriched in the selenium group are visualized in Figure 2. Two main clusters of gene sets were identified and were classified as A) wound healing, cellular adhesion and extracellular matrix interactions and B) inflammation and immunity. All of the gene sets in these two clusters were considered ‘negative’ which implies that genes in these gene-sets were down-regulated after the intervention with selenium. Furthermore, expression of genes involved in cellular adhesion, inflammation, extracellular matrix interactions and chemokine signaling was down-regulated after intervention with selenium. For the placebo group, gene sets referring to inflammation, infection, hemostasis and cellular adhesion were enriched and considered ‘positive’ after the intervention (data not presented), which again points towards the opposite effects on gene expression found after the intervention with selenium versus placebo.

Based on the IPA Upstream Regulator Analysis, we identified regulators that may have been responsible for the observed expression changes. Predicted upstream regulators were considered relevant if the z-score was below -2 or above 2 and if the p-value of overlap was <0.05. The top-5 (based on z-score) of the predicted activated and inhibited regulators is presented in Table 2. Transforming growth factor β1 (*TGFB1*) was identified as an upstream regulator predicted to be inhibited in the selenium group and activated in the placebo group (p-value of overlap 3.70E-06 and 4.35E-12, respectively).

**Table 2:** The top-5 of the predicted activated and inhibited upstream regulators with the highest and lowest z-scores according to IPA Upstream Regulator Analysis

	Fold change after intervention	Molecule type	Predicted activation state	Activation z-score	p-value of overlap
**Selenium**					
TNF	-1.1	cytokine	Inhibited	-3.85	1.94E-03
PDGF BB	NA	complex	Inhibited	-2.56	3.39E-05
TP53	1.1	transcription regulator	Inhibited	-2.32	4.44E-03
TGFB1	-1.2	growth factor	Inhibited	-2.28	3.70E-06
STAT5a/b	NA	group	Inhibited	-2.24	1.93E-03
TAB1	-1.0	enzyme	Activated	2.00	1.39E-02
SPDEF	1.4	transcription regulator	Activated	2.16	3.97E-02
MAP3K7	-1.0	kinase	Activated	2.22	1.02E-02
WISP2	-1.2	growth factor	Activated	2.45	3.16E-03
**Placebo**					
COL18A1	1.1	other	Inhibited	-3.77	1.94E-07
SPDEF	-1.3	transcription regulator	Inhibited	-2.95	3.75E-04
NEUROG1	-1.0	enzyme	Inhibited	-2.83	3.85E-08
JAG2	1.1	complex	Inhibited	-2.45	1.30E-02
IL1RN	1.2	transcription regulator	Inhibited	-2.38	1.66E-03
IFNG	NA	cytokine	Activated	4.89	1.03E-12
PDGF BB	NA	complex	Activated	5.48	9.59E-20
TGFB1	1.3	growth factor	Activated	5.70	4.35E-12
TGM2	1.8	enzyme	Activated	6.10	2.79E-09
TNF	1.1	cytokine	Activated	6.94	7.97E-29

For calculation of the z-score IPA included information about the up- or down-regulated status of the genes in the dataset. For this analysis, the expression of genes was considered up- or down-regulated if the p-value for the within (after intervention versus baseline) and between group (changes in selenium versus placebo) comparisons was below p<0.05. Upstream regulators in the top-5 of the highest and lowest z-scores are only presented if their p-value of overlap was below 0.05.

Abbreviations: *IPA* Ingenuity Pathway Analysis, NA not available

Focusing on the network of *TGFB1* and a selection of other predicted upstream regulators in the selenium group, i.e. the SAM pointed domain containing ETS transcription factor (*SPDEF*) and WNT1 inducible signaling pathway protein 2 (*WISP2*), showed some common targets that are directly or indirectly controlled by these regulators (Supplementary Figure 3). Vimentin (*VIM*), E-cadherin (*CDH1*), OB-cadherin (*CDH11*) and connective tissue growth factor (*CTGF*) are all considered to be regulated by *TGFB1*, *SPDEF* and *WISP2*. Interestingly, both *TGFB1* as well as the down-stream genes *CDH1*, *CDH11*, *VIM* and *CTGF* are suggested to be involved in the epithelial-to-mesenchymal transition (EMT). EMT is a process characterized by the transition of an epithelial phenotype towards a mesenchymal phenotype and is implicated in embryogenesis, inflammation, wound healing, as well as cancer progression and metastasis.

**Figure 3:**
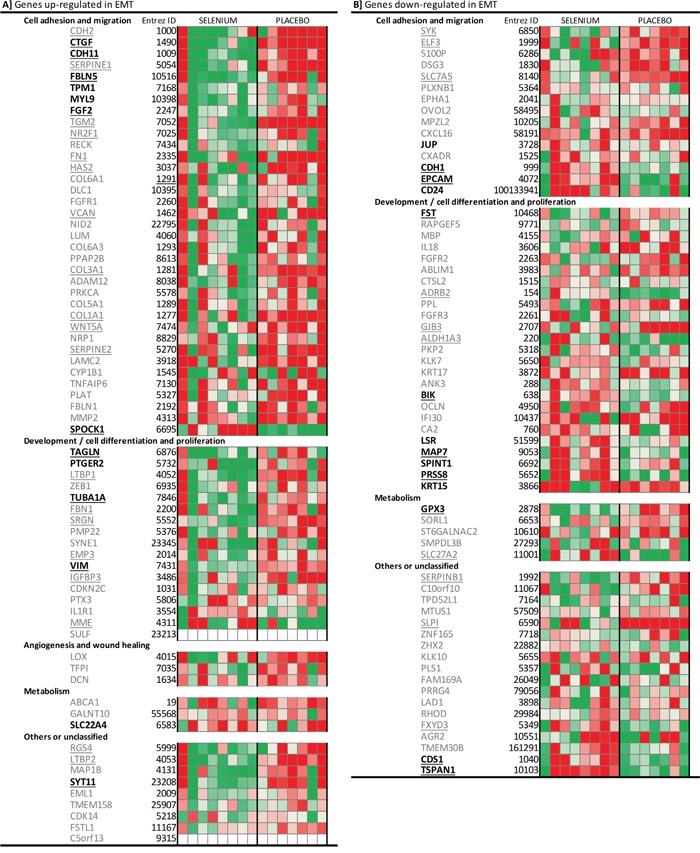
Heatmap of the individual gene expression changes of 130 EMT-core genes in participants receiving selenium or placebo According to literature [reference 15], **panel A** represent genes that are up-regulated during EMT, while **panel B** includes genes that are down-regulated during EMT. Changes in gene expression are presented as signal-log-ratios (green; SLR ≤ -0.5 to red; SLR ≥ 0.5). For genes with bold names, the expression changed after the intervention with selenium (Limma p-value <0.05). Underlined genes had expression changes that are significantly different between the selenium and placebo group (Limma p-value <0.05). Abbreviations: *EMT* epithelial-to-mesenchymal transition, *SLR* Signal-Log-Ratio.

Based on literature [15], we obtained an EMT-core gene list of 130 genes that are implicated in EMT and cancer progression. Gene expression changes after intervention for all our participants were plotted amongst this EMT-core list (Figure 3). Overall, the intervention with selenium resulted in down-regulated expression of genes known to be up-regulated during EMT. More specifically, expression of the ‘mesenchymal markers’ *FN1* (fibronectin), *VIM*, and *CDH11* was down-regulated, while ‘epithelial markers’, such as *CDH1*, *SDC1* (syndecan-1) and *EPCAM* (epithelial cell adhesion molecule), were up-regulated after intervention with selenium.

**Figure 4:**
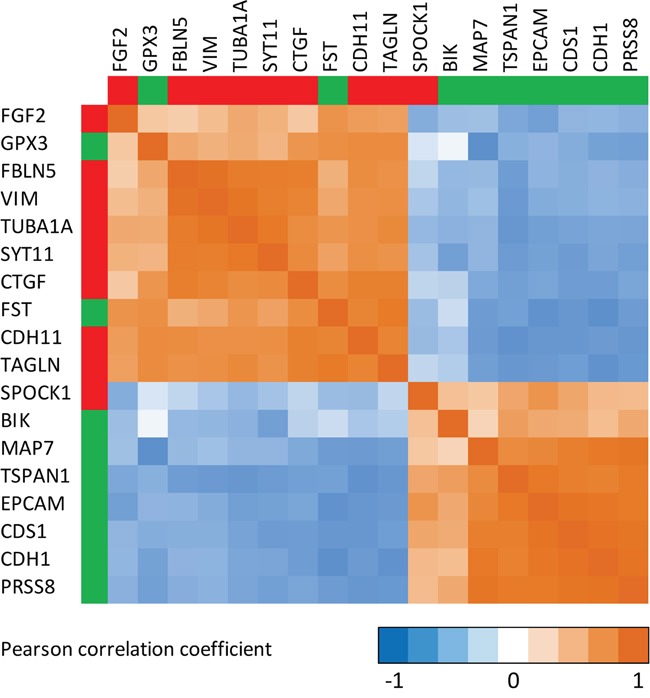
Correlation matrix showing the correlations between the expression changes for genes involved in epithelial-to-mesenchymal transition (EMT) Calculation of the Pearson correlation coefficient is based on the individual signal-log-ratios representing the expression changes after the intervention with selenium. Only genes from the EMT-core [15] list for which the expression changed after the intervention with selenium and differed from the placebo group (Limma p-value <0.05) are presented. Green legends besides the genes indicate that these genes are commonly down-regulated during EMT, while genes presented in red are commonly up-regulated during EMT [15].

**Figure 5:**
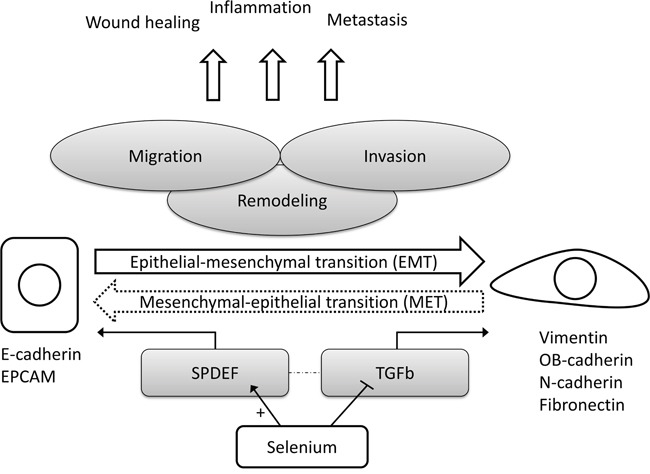
Simplified hypothetical model for the suggested effects of selenium on epithelial-to-mesenchymal transition in prostate tissue Abbreviations: *EPCAM* epithelial cell adhesion molecule, *EMT* epithelial-to-mesenchymal transition, *MET* mesenchymal-to-epithelial transition, *SPDEF* SAM pointed domain containing ETS transcription factor, *TGFb* transforming growth factor beta.

In order to explore the consistency and robustness of our findings, we correlated the selenium-induced expression changes of all statistically significant (in within-, and between-group comparisons) EMT-related genes amongst each other. The corresponding Pearson correlation matrix for selenium (Figure 4) showed that expression changes of genes that are known to be up-regulated during EMT (i.e. mesenchymal markers) were inversely correlated to the expression changes of genes down-regulated during EMT (i.e. epithelial markers). A simplified hypothetical model for the suggested effects of selenium on EMT in the prostate is presented in Figure 5.

## DISCUSSION

Our study demonstrated that a 5-week intervention with selenium was associated with changes in expression of genes implicated in EMT in non-malignant prostate tissue. EMT is characterized by the transition of an epithelial towards mesenchymal phenotype with its accompanying properties such as the ability to migrate and invade [16, 17]. Based on our findings, we hypothesized that selenium may be able to prevent, inhibit or reverse the transition of the epithelial to the mesenchymal phenotype. Expression of numerous well-established epithelial cell markers was increased, while expression of mesenchymal markers was decreased after intervention with selenium. The observation that expression changes for epithelial versus mesenchymal markers were inversely correlated implies that our findings were consistent and were not likely resulting from chance findings.

EMT plays an important role during embryogenesis, inflammation and wound healing as cellular migration and invasion facilitates organ development, tissue (re)modelling and regeneration [17, 18]. In parallel with the reported expression changes for genes implicated in EMT, expression of genes involved in these processes was affected after the intervention with selenium as well. A possible, but speculative, explanation for the pronounced inflammatory gene expression profile observed in the placebo group may be a persisting inflammatory response resulting from the first series of prostate biopsies. Interestingly, this inflammatory expression profile was not observed in the selenium group, which points towards a possible anti-inflammatory effect of selenium in the prostate. It should be noted that the median, but not mean, time between the repeated collection of prostate biopsies tended to be slightly shorter for participants in the selenium versus placebo group. The time between the biopsies for the individual participants was [32, 32, 32, 35, 63, 91, 98, 112 days] for the selenium group and [35, 36, 63, 65, 72, 112, 112 days] for the placebo group, which did not reveal any systematic differences between the groups.

The intervention with selenium was also associated with expression changes of genes involved in wound healing, cellular adhesion and extracellular matrix interactions. Based on our GSEA results, we identified a cluster of gene sets related to wound healing which were down-regulated after the intervention with selenium. The pronounced effects hinting towards wound healing in the placebo group may again refer to repair and remodeling of prostate tissue resulting from the (repeated) biopsies. Interestingly, in the selenium group expression of genes involved in wound healing was down-regulated. Based upon these data, we carefully hypothesized that selenium may contribute to accelerated or improved wound healing in the prostate.

So far, the role of selenium in wound healing has not been extensively described. Few small clinical studies or animal experiments suggest that selenium may improve and accelerate wound healing under various conditions [19, 20]. One of the potential key players implicated in wound healing is the gap junction protein connexin43 (Cx43) encoded by the *GAJ1* gene [21]. Experimental down-regulation of Cx43 at wound sites resulted in an improved rate and quality of healing [22]. Our intervention with selenium was also associated with down-regulated expression of *GAJ1*/Cx43 as well as *GJC1*/Cx45, whereas in the placebo group the expression of these connexins did not change. Interestingly, increased expression of *GAJ1*/Cx43 has also been linked to metastatic behavior of prostate cancer cells [23, 24].

The potential mechanisms underlying the effect of selenium on EMT, with or without a direct relationship to wound healing and inflammation, have not been described in detail previously. It has consistently been shown that transforming growth factor β1 (*TGFB1*) is a major inducer of EMT [17, 25] and is also involved in inflammation and wound healing [18, 26]. In our study, the expression of *TGFB1* is significantly down-regulated after the intervention with selenium, while up-regulated in the placebo group. *TGFB1* was also one of the main predicted upstream transcriptional regulators. Changes in protein expression of TGFbeta in serum of elderly men after selenium supplementation have been previously described [27].

We also identified the sterile alpha motif (SAM) pointed domain-containing ETS transcription factor (*SPDEF*) as an upstream transcriptional regulator. *SPDEF* is highly expressed in normal prostatic epithelial tissue [28, 29]. Previous studies showed that *SPDEF* is required for *CDH1* expression in prostate cancer cells [30] and that *SPDEF* is a downstream target of *TGFB1* [31]. Our intervention with selenium was associated with increased *SPDEF* expression which is in line with the epithelial phenotype and the increase in *CDH1* expression. In previous studies, it was consistently shown that SPDEF inhibits cancer cell migration *in vitro* [31, 32] as well as prostate cancer metastasis *in vivo* [33]. Loss of SPDEF was associated with occurrence of aggressive high-grade prostate cancer [32, 34]. Methylseleninic acid (MSA), a synthetic selenium compound, has been shown to induce expression of *SPDEF* in prostate cancer cells [34], which is in line with the findings of our study.

To the best of our knowledge, the direct role of selenium in the regulation of EMT and related processes in the prostate has not been described in detail before. From *in vitro* work, it is known that the effects of selenium on EMT-related gene expression as well as migratory capacity strongly depend on the selenium compound used, the experimental conditions and the cell types or tissues studied [35–40]. Data from human studies assessing the effects of selenium on gene expression profiles in prostate tissue are scarce. Tsavachidou and colleagues examined gene expression in distinct anatomical zones and cell types of the prostate after a 3-6 week intervention with *L*-selenomethionine [14]. Prostate tissue was collected at a single time point after the intervention period [14]. We collected prostate tissue before and after intervention, which allowed us to compare changes in gene expression within individuals. This aspect can be considered a major strength of our study, because variation due to inter-individual differences did not hinder the interpretation of the microarray data. Furthermore, due to the repeated sample collection, we were able to detect changes in gene expression that may not be directly attributed to the intervention with selenium, such as responses to the biopsy or other clinical procedures.

The marginal baseline selenium status of the participants may also be considered a strength of our study. In the NPC trial, specifically the participants with relatively low selenium status at baseline (selenium <123.2 μg/L) seemed to benefit from the intervention with selenized yeast [2]. Baseline selenium levels of the participants of the SELECT trial were relatively high (median 135 μg/L) [6]. The median selenium level of our participants was 81 μg/L and increased to 185 μg/L after the intervention. Based on experimental studies in dogs and a number of observational studies in humans, a U-shaped dose response curve for selenium status and several health outcomes was suggested [10, 41, 42]. While overlaying the U-shaped dose response curve with our data, it seemed that our participants started with a ‘low status’ and ended with an ‘optimal to high selenium status’. One should, however, carefully interpret these findings, since it is not possible to draw conclusions about beneficial health effects of selenium in the current setting. Furthermore, it should be mentioned that the optimal range of selenium intake and status is narrow and strongly depends on various factors such as intake of various selenium compounds [43], genotype [44, 45], and metabolic capacity [10].

A limitation of our study refers to the relatively small number of participants. Recruitment of participants was complicated inherent to the design of our study which required prostate sample collection at two consecutive time points and the unanticipated negative outcome of the SELECT trial. To what extent sample size affects the power to detect gene expression changes is difficult to assess for these data-driven expression profiling studies. At baseline, differences in gene expression exist between the selenium and placebo group (n=2624 genes), which may be attributable to unanticipated variation or outliers and the relatively small sample size. A subset of these genes was also differently expressed upon the intervention with selenium (n=437 genes, 17%). We cannot exclude the possibility that these baseline differences to some extent affected the responses observed. However, despite substantial variation and differences between the groups at baseline, gene expression changes were highly consistent within the groups after intervention. Furthermore, we examined effects of selenium supplementation at a transcriptional level and therefore we cannot rule out any potential post-transcriptional effects that may determine activity and functionality of other potential regulators, such as the selenoproteins.

For the current study, we collected non-malignant tissue from the transition zone of the prostate. Approximately 20% of the prostatic adenocarcinomas arise from this zone, in comparison to 70-80% from the peripheral zone [46]. The transition zone and the peripheral zones have been shown to differ in gene expression profiles [46], which may have consequences for the functional implications of our findings in other zones of the prostate. Limited amounts of tissue and common clinical practices precluded the assessment of selenium levels in the prostate or serum PSA levels after intervention, respectively. Other studies, however, have already shown that short-term (2-6 weeks) supplementation with selenized yeast or L-selenomethionine resulted in increased levels of selenium in the prostate [13, 47, 48].

In conclusion, our explorative data imply that selenium is associated with the regulation of genes involved in cellular migration, invasion, tissue remodeling and the immune response in non-malignant tissue collected from the transition zone of the prostate. These processes are implicated in EMT and related events such as inflammation, wound healing and cancer progression. Taken altogether, these results suggest a preventive effect of selenium on prostate cancer progression rather than on prostate cancer development and future studies are warranted to confirm this hypothesis.

## MATERIALS AND METHODS

### Participants and design of the study

For this study, we collected non-malignant prostate tissue before and after a short-term intervention with selenized yeast or yeast placebo. We recruited participants from the Radboud university medical center, an academic tertiary referral center in Nijmegen, The Netherlands. Men scheduled for diagnostic prostate biopsies, and subsequent treatment with radical prostatectomy (RP) or radiotherapy (RT) for prostate cancer, were invited for this study. Also, men scheduled for re-biopsies because of high-grade prostatic intraepithelial neoplasia (HGPIN) were eligible for inclusion. For practical and ethical reasons, the men included in this study were all at high risk for prostate cancer or diagnosed with prostate cancer, as collection of prostate tissue for the study had to be combined with standard invasive clinical procedures. However, for all participants only non-malignant prostate tissue, verified by histological examinations, was considered for the analyses.

Exclusion criteria were current use of dietary supplements providing more than the recommended daily allowance of 55 μg selenium per day, any malignancy in the preceding five years (except for non-melanoma skin cancer), current hepatic or renal disease or inflammatory bowel disease, and neo-adjuvant therapies for prostate cancer.

Overall, 23 participants were eligible for participation in the intervention study (Supplementary Figure 1). Before start of the intervention, prostate tissue, blood samples and toenail clippings were collected and body weight and height were measured. Furthermore, participants filled out a baseline questionnaire on sociodemographic characteristics, medical history and use of dietary supplements.

### Intervention

Participants were randomly assigned using a permuted-block design (blocks of four participants) to take 300 μg selenium per day in the form of selenized yeast (SelenoPrecise®, Pharma Nord, Denmark) or a placebo (non-selenized yeast, Pharma Nord, Denmark). The non-selenized yeast tablets were based on yeast grown on selenium-deficient medium and smell, taste and appearance did not differ from the intervention tablets. Both intervention and placebo tablets were previously used in the PRECISE Trial pilot studies [49–51] and have been shown to have a stable batch-to-batch profile with L-selenomethionine (∼81%) as the most abundant selenium compound in the intervention tablets [51, 52]. Compliance was assessed by checking research diaries, counting returned tablets and measuring serum selenium levels before and after intervention. The intervention period had an intended duration of five weeks and was depending on the time between enrollment and final treatment or re-biopsy. The intervention period of five weeks was chosen because this resembles the average time between diagnostic biopsies and treatment by RP. During the intervention period, participants were asked to take daily supplements with selenium or placebo. At the end of the intervention period, prostate tissue and blood samples were collected for 22 participants. Sample collection for one participant failed because of logistic reasons. All participants returned remaining pills and their completed research diaries in which details concerning the use of study supplements were registered. The institutional review board (CMO Regio Arnhem-Nijmegen) approved the design of the study and all participants provided written informed consent. The trial was registered at clinicaltrials.gov with identifier NCT00446901.

### Selenium analyses

For serum selenium analyses, blood was collected into 10-ml serum tubes (Becton Dickinson B.V.). Serum was collected after centrifugation and stored at -20°C until analyses. Serum selenium levels were measured using an atomic absorption spectrometer (model 4100ZL, PerkinElmer) coupled with a graphite furnace and using Zeeman background correction [53]. The detection limit for this method was 0.10 μmol/L. For each analytical run, a series of standards (CertiPur® AAS standards, no. 1197960100, Merck Chemicals, Germany) and a control (Pathonorm-High^TM^, SERO AS, Norway) were included. All samples were analyzed in triplicate.

Baseline toenail selenium levels, indicative of a long-term selenium intake and status [54], were assessed using Instrumental Neutron Activation Analyses (INAA) at the Reactor Institute of Delft University, the Netherlands, as described previously [55].

### Collection of prostate tissue

Prostate tissue was collected using an 18-gauge biopsy needle (Bard Biopsy Systems) during regular 10-core prostate needle biopsy series guided by transrectal ultrasound (TRUS) or with a biopsy during RP. In both cases, the study biopsy was taken from the superior, ventral region of the left lobe of the prostate, which is specified as the transition zone. The study biopsy was directly embedded in Optimal Cutting Temperature (O.C.T.) Compound (Sakura Finetek Europe B.V.) and snapfrozen in liquid nitrogen. All samples were stored at -80°C.

### Histology

The biopsies embedded in O.C.T. Compound were sectioned at -20°C. Representative sections of 5 μm were used for histological examinations; at least two sections were used for a Haematoxilin-Eosin (HE) staining. The remainder of the biopsy was sectioned at 20 μm and used for RNA extraction. All HE-stained slides were reviewed for malignancy, HGPIN and inflammation by an expert uropathologist.

### RNA extraction

Total RNA was extracted from the sectioned prostate biopsies using TRIzol Reagent according to the manufacturer’s instructions (Invitrogen). Isolated RNA was purified using RNeasy Micro columns (Qiagen). RNA integrity (RNA 6000 Nanochips for the Agilent 2100 Bioanalyzer, Agilent Technologies) and total RNA yield (Nanodrop ND 1000, Nanodrop Products) were assessed for all samples. The mean RNA integrity (RIN) score was 8.2 (standard deviation: 0.66). Prostate biopsies with inadequate RNA yield (<20 ng/μl, n=1) or histological evidence of adenocarcinoma (n=6) were excluded, leaving biopsies of 15 participants available for microarray analyses (n=8 selenium, n=7 placebo) (Supplementary Figure 1).

### Microarray analyses

A total of 30 RNA samples were processed for microarray analyses. Briefly, 100 ng of total RNA per sample was converted to cDNA, labeled using an Ambion WT Expression kit and hybridized to Affymetrix GeneChip Human Gene 1.0 ST Arrays. Probe sets were redefined according to Dai et al. [56] using the remapped chip description files (CDF) version 14.1.1 based on the Entrez Gene database. The normalized signal intensities were expressed as Robust Multichip Average (RMA) expression values [57]. Genes with RMA expression values >20 in at least 4 arrays were considered as expressed in prostate tissue and were selected for further analyses. Ratios of the log(base2) transformed intensity signals (signal-log-ratios, SLR) were used to compare the individual microarray data before and after the intervention. Changes in gene expression within each of the groups were considered statistically significant if the p-value derived from the linear model (Limma: Linear Models for Microarray Data) [58] with pairwise comparisons and empirical Bayesian correction was <0.05. Differentially changed genes between the two groups were also identified using the Limma models with Bayesian correction (Limma p-value <0.05). Microarray data are available from the Gene Expression Omnibus (GEO) repository with accession number GSE77959.

### Biological pathway analyses

Regulated pathways were identified through the use of IPA (version 18030641, Ingenuity® Systems, www.ingenuity.com). For these analyses, expression of genes was considered up- or down-regulated if the p-value for the within- and between-group comparisons was <0.05. Canonical pathways with a p-value of <0.01 in the Fisher’s exact test were considered as significant to the data. IPA Upstream Regulator Analysis was used to predict common regulators that may be responsible for the observed changes in gene expression. Prediction of regulators is based on two parameters; the z-score and the p-value of overlap. The z-score reflects the consistency of the gene expression patterns of the downstream genes in the dataset, whereas the p-value of overlap is calculated by a Fisher’s exact test for the overlap between the genes in a dataset (expected) and the genes actually regulated by the potential transcriptional regulator (observed).

In order to further explore the biological relevance and consistency of our findings, we also conducted Gene Set Enrichment Analyses (GSEA, http://www.broadinstitute.org/gsea/index.jsp) [59] for the changes in gene expression after the intervention with either selenium or placebo. Cytoscape 2.8.2 [60] was used to visualize the GSEA results in an enrichment map [61]. Gene sets with a False Discovery Rate (FDR) q-value of <0.01 were considered enriched to the data.

Gitools software was used to construct a correlation matrice showing the Pearson correlation coefficients for the individual expression changes expressed as signal-log-ratios of selected genes [62].

### Statistical analysis

Since levels of selenium after intervention, duration of intervention and a number of other participants’ characteristics were not normally distributed, data were summarized as median and interquartile ranges or numbers and percentages. Baseline serum and toenail selenium levels were compared between the selenium group and the placebo group using the Mann-Whitney U test. Serum selenium levels after intervention were compared to baseline values using the Wilcoxon Signed Rank test. All statistical tests were two-sided and p-values below <0.05 were considered as statistically significant. Statistical Package for Social Sciences (SPSS version 19, Chicago, Illinois) was used for all analyses unless otherwise stated.

## SUPPLEMENTARY MATERIALS FIGURES AND TABLES




