# Supplementary material for: A short-term intervention with selenium affects expression of genes implicated in the epithelial-to-mesenchymal transition in the prostate
Source: Oncotarget. 2017 Jan 6;8(6):10565–79. doi: 10.18632/oncotarget.14551 (PMC5354681; doi:10.18632/oncotarget.14551)
Supplement: Supplementary file 1 [file oncotarget-08-10565-s001.pdf]

A short-term intervention with selenium affects expression of genes implicated in the epithelial-to-mesenchymal transition in the prostate

SUPPLEMENTARY FIGURES AND TABLE

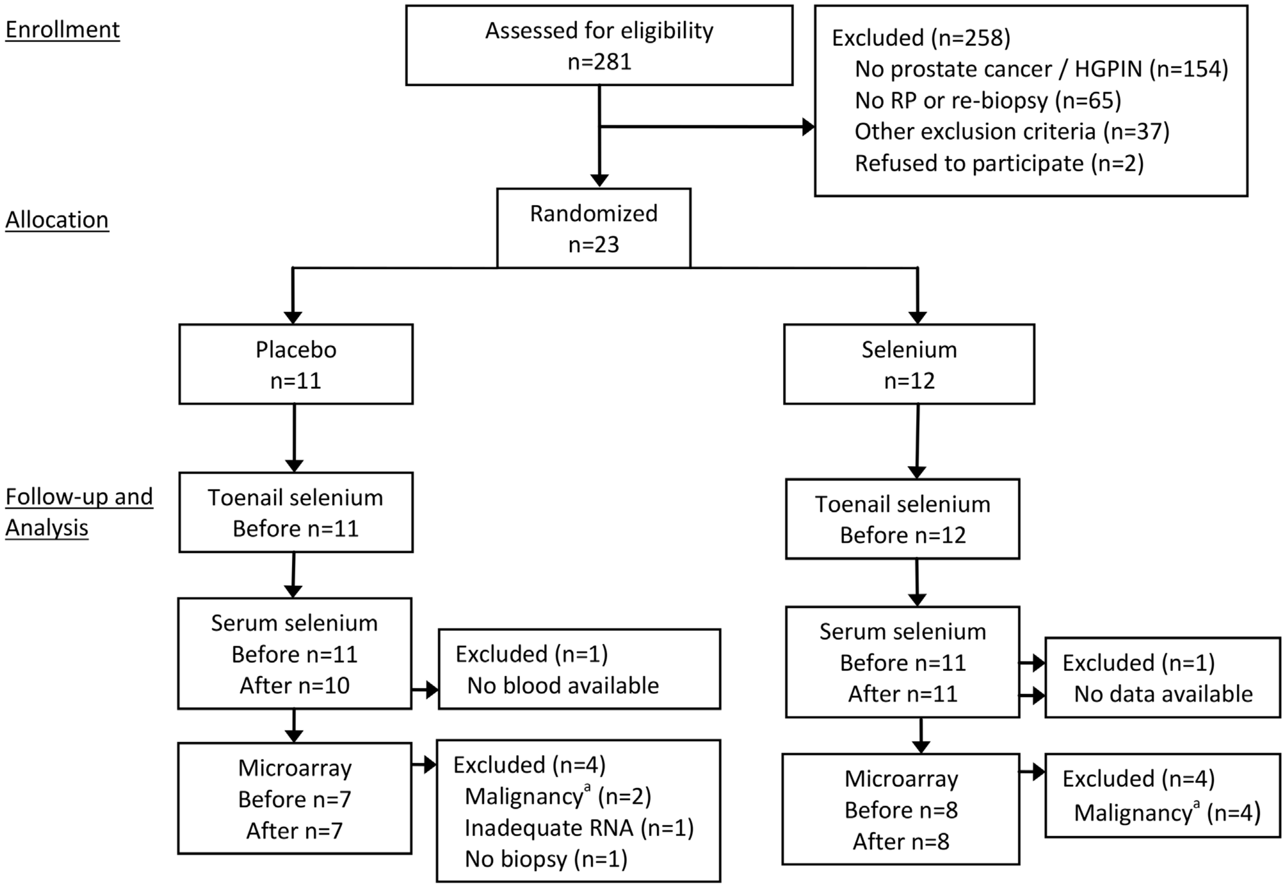

[a]Malignancy found in the study biopsy.
Abbreviations: RP radical prostatectomy, HGPIN high-grade prostatic intraepithelial neoplasia.

Supplementary Figure S1: Study flow chart.

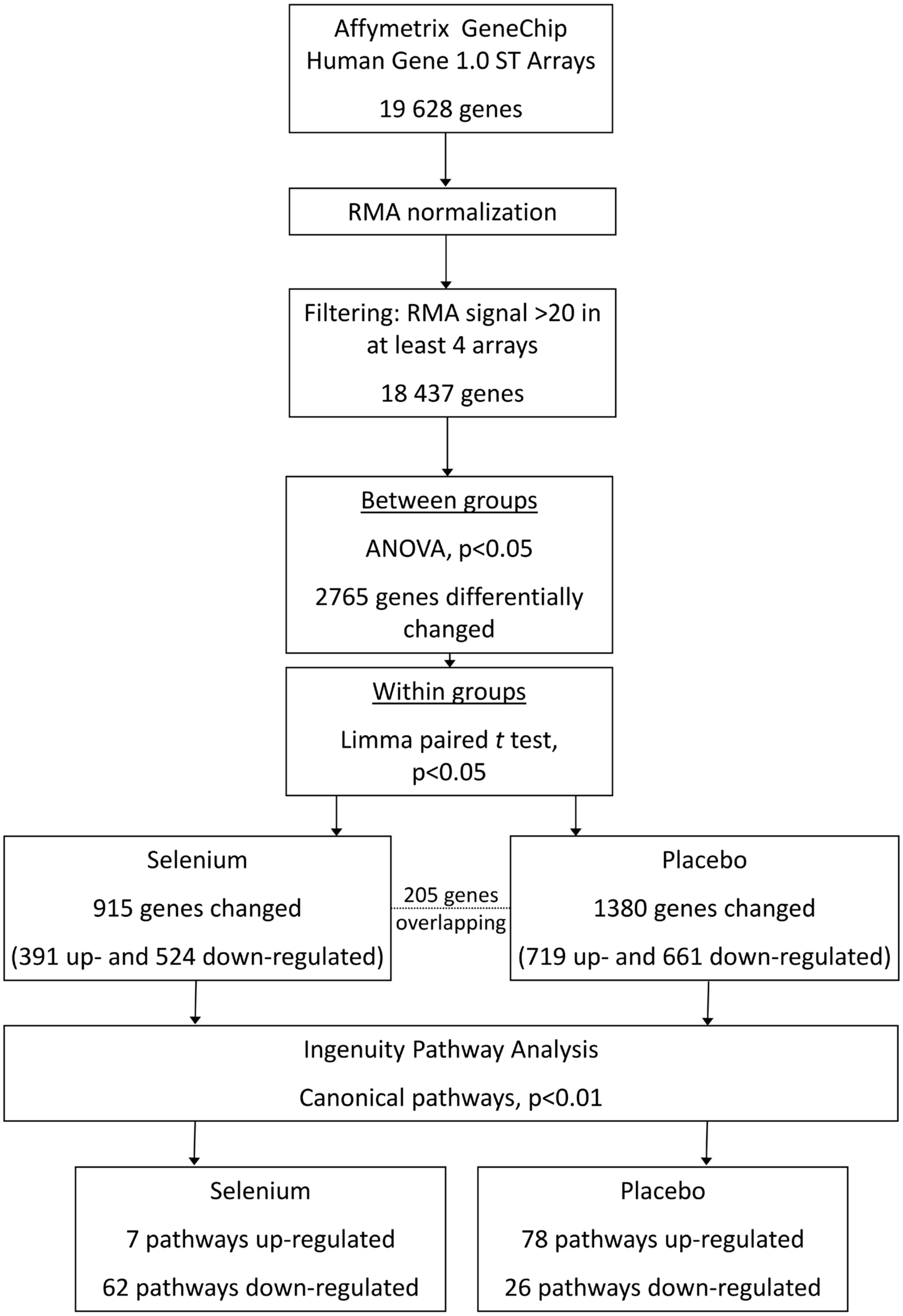

Supplementary Figure S2: Flow diagram of the gene selection for the microarray analyses. Abbreviations: ANOVA analysis of variance, RMA Robust Multichip Average.

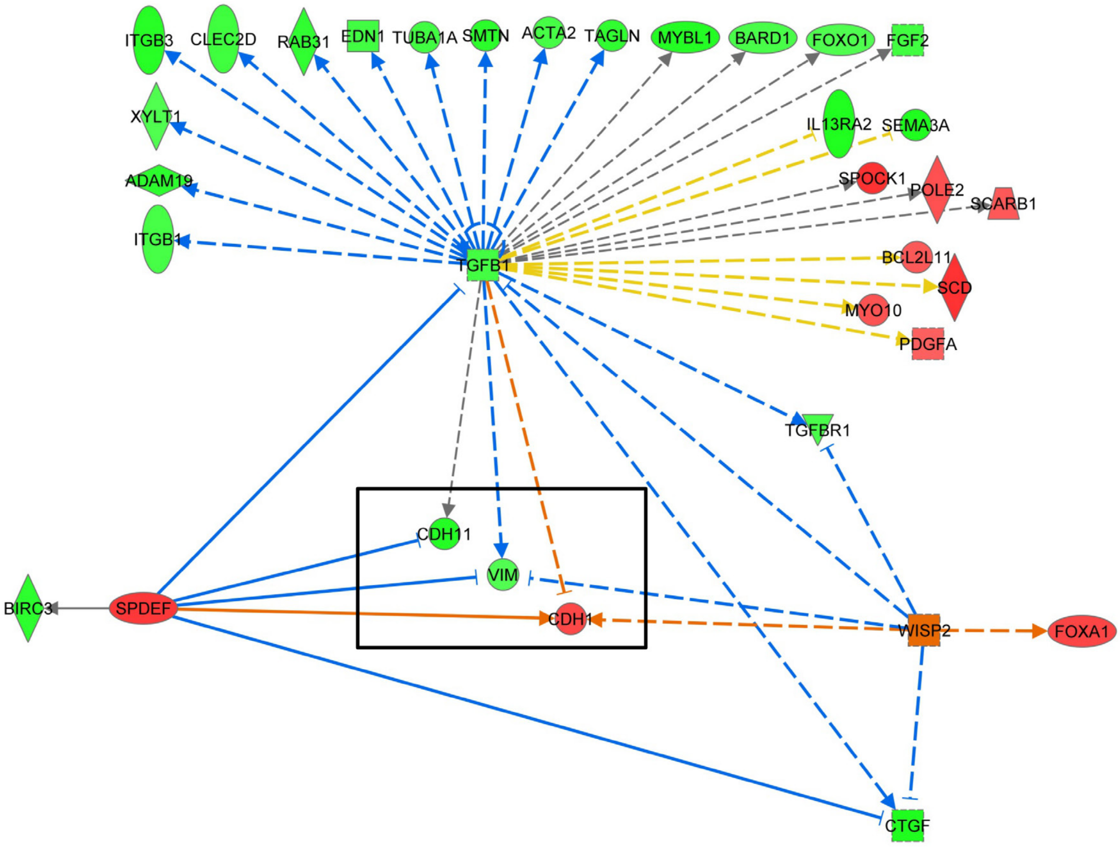

Supplementary Figure S3: IPA-derived network for the predicated upstream regulators in the selenium group. This network includes the predicted upstream regulators TGFB1, WISP2, SPDEF and their downstream regulated genes according to the IPA Upstream Regulator Analysis. A dashed line represents an indirect relationship, while a solid line refers to a direct relationship between genes or molecules. The color of the lines indicates whether the predicted relationship leads to inhibition (blue), activation (orange), is not predicted (grey) or is inconsistent with the findings (yellow). The color of the downstream genes indicates whether expression is up-regulated (red) or down-regulated (green) after the intervention with selenium. The common epithelial (CDH1, E-cadherin) and mesenchymal markers (VIM, vimentin and CDH11, OB-cadherin) are presented in the box.

Supplementary Table S1: Genes with most pronounced expression changes within the selenium or placebo group

See Supplementary File 1
