# Supplementary material for: A short-term intervention with selenium affects expression of genes implicated in the epithelial-to-mesenchymal transition in the prostate
Source: Oncotarget. 2017 Jan 6;8(6):10565–79. doi: 10.18632/oncotarget.14551 (PMC5354681; doi:10.18632/oncotarget.14551)
Supplement: Supplementary file 2 [file oncotarget-08-10565-s002.docx]

Supplementary table S1

| **SELENIUM** | | | | |
| --- | --- | --- | --- | --- |
| **Entrez ID** | **Gene symbol** | **Gene name** | **Fold change** | **p-value** |
| 4057 | LTF | lactotransferrin | -3.2 | 0.019 |
| 11075 | STMN2 | stathmin-like 2 | -2.2 | 0.033 |
| 8549 | LGR5 | leucine-rich repeat containing G protein-coupled receptor 5 | -2.2 | 0.017 |
| 8516 | ITGA8 | integrin, alpha 8 | -2.0 | 0.007 |
| 84189 | SLITRK6 | SLIT and NTRK-like family, member 6 | -1.9 | 0.002 |
| 389658 | FAM150A | family with sequence similarity 150, member A | -1.9 | 0.027 |
| 3624 | INHBA | inhibin, beta A | -1.8 | 0.023 |
| 81610 | FAM83D | family with sequence similarity 83, member D | -1.8 | 0.012 |
| 387758 | FIBIN | fin bud initiation factor homolog (zebrafish) | -1.7 | 0.042 |
| 10371 | SEMA3A | sema domain, immunoglobulin domain (Ig), short basic domain, secreted, (semaphorin) 3A | -1.7 | 0.012 |
| 343450 | KCNT2 | potassium channel, subfamily T, member 2 | -1.7 | 0.006 |
| 9358 | ITGBL1 | integrin, beta-like 1 (with EGF-like repeat domains) | -1.6 | 0.034 |
| 3598 | IL13RA2 | interleukin 13 receptor, alpha 2 | -1.6 | 0.017 |
| 50507 | NOX4 | NADPH oxidase 4 | -1.6 | 0.019 |
| 5139 | PDE3A | phosphodiesterase 3A, cGMP-inhibited | -1.6 | 0.021 |
| 157869 | C8orf84 | chromosome 8 open reading frame 84 | -1.6 | 0.019 |
| 57575 | PCDH10 | protocadherin 10 | -1.6 | 0.009 |
| 26002 | MOXD1 | monooxygenase, DBH-like 1 | -1.6 | 0.013 |
| 2487 | FRZB | frizzled-related protein | -1.6 | 0.010 |
| 28983 | TMPRSS11E | transmembrane protease, serine 11E | -1.6 | 0.010 |
| 4915 | NTRK2 | neurotrophic tyrosine kinase, receptor, type 2 | -1.6 | 0.021 |
| 10129 | FRY | furry homolog (Drosophila) | -1.6 | 0.029 |
| 27295 | PDLIM3 | PDZ and LIM domain 3 | -1.6 | 0.009 |
| 10631 | POSTN | periostin, osteoblast specific factor | -1.6 | 0.050 |
| 9369 | NRXN3 | neurexin 3 | -1.6 | 0.026 |
| 27129 | HSPB7 | heat shock 27kDa protein family, member 7 (cardiovascular) | -1.6 | 0.009 |
| 953 | ENTPD1 | ectonucleoside triphosphate diphosphohydrolase 1 | -1.5 | 0.001 |
| 2823 | GPM6A | glycoprotein M6A | -1.5 | 0.044 |
| 91624 | NEXN | nexilin (F actin binding protein) | -1.5 | 0.003 |
| 7111 | TMOD1 | tropomodulin 1 | -1.5 | 0.006 |
| 8842 | PROM1 | prominin 1 | -1.5 | 0.028 |
| 5648 | MASP1 | mannan-binding lectin serine peptidase 1 (C4/C2 activating component of Ra-reactive factor) | -1.5 | 0.003 |
| 2669 | GEM | GTP binding protein overexpressed in skeletal muscle | -1.5 | 0.005 |
| 5350 | PLN | phospholamban | -1.5 | 0.002 |
| 23213 | SULF1 | sulfatase 1 | -1.5 | 0.015 |
| 11259 | FILIP1L | filamin A interacting protein 1-like | -1.5 | 0.006 |
| 1490 | CTGF | connective tissue growth factor | -1.5 | 0.016 |
| 254228 | FAM26E | family with sequence similarity 26, member E | -1.5 | 0.004 |
| 79750 | ZNF385D | zinc finger protein 385D | -1.5 | 0.025 |
| 57188 | ADAMTSL3 | ADAMTS-like 3 | -1.5 | 0.046 |
| 7881 | KCNAB1 | potassium voltage-gated channel, shaker-related subfamily, beta member 1 | -1.5 | 0.000 |
| 407006 | MIR221 | microRNA 221 | -1.5 | 0.014 |
| 2273 | FHL1 | four and a half LIM domains 1 | -1.5 | 0.016 |
| 6401 | SELE | selectin E | -1.5 | 0.026 |
| 9595 | CYTIP | cytohesin 1 interacting protein | -1.5 | 0.015 |
| 58189 | WFDC1 | WAP four-disulfide core domain 1 | -1.5 | 0.003 |
| 627 | BDNF | brain-derived neurotrophic factor | -1.5 | 0.026 |
| 100507025 | LOC100507025 | hypothetical LOC100507025 | 1.5 | 0.024 |
| 161436 | EML5 | echinoderm microtubule associated protein like 5 | 1.5 | 0.043 |
| 8349 | HIST2H2BE | histone cluster 2, H2be | 1.5 | 0.028 |
| 54566 | EPB41L4B | erythrocyte membrane protein band 4.1 like 4B | 1.5 | 0.003 |
| 3817 | KLK2 | kallikrein-related peptidase 2 | 1.5 | 0.043 |
| 57512 | GPR158 | G protein-coupled receptor 158 | 1.5 | 0.036 |
| 4824 | NKX3-1 | NK3 homeobox 1 | 1.5 | 0.016 |
| 51109 | RDH11 | retinol dehydrogenase 11 (all-trans/9-cis/11-cis) | 1.5 | 0.035 |
| 84084 | RAB6C | RAB6C, member RAS oncogene family | 1.5 | 0.022 |
| 131034 | CPNE4 | copine IV | 1.5 | 0.032 |
| 57475 | PLEKHH1 | pleckstrin homology domain containing, family H (with MyTH4 domain) member 1 | 1.5 | 0.006 |
| 55503 | TRPV6 | transient receptor potential cation channel, subfamily V, member 6 | 1.5 | 0.015 |
| 54898 | ELOVL2 | ELOVL fatty acid elongase 2 | 1.5 | 0.028 |
| 285175 | UNC80 | unc-80 homolog (C. elegans) | 1.5 | 0.008 |
| 2786 | GNG4 | guanine nucleotide binding protein (G protein), gamma 4 | 1.5 | 0.020 |
| 51280 | GOLM1 | golgi membrane protein 1 | 1.5 | 0.014 |
| 440689 | HIST2H2BF | histone cluster 2, H2bf | 1.5 | 0.001 |
| 11013 | TMSB15A | thymosin beta 15a | 1.5 | 0.010 |
| 6695 | SPOCK1 | sparc/osteonectin, cwcv and kazal-like domains proteoglycan (testican) 1 | 1.5 | 0.005 |
| 4986 | OPRK1 | opioid receptor, kappa 1 | 1.6 | 0.036 |
| 1803 | DPP4 | dipeptidyl-peptidase 4 | 1.6 | 0.013 |
| 6296 | ACSM3 | acyl-CoA synthetase medium-chain family member 3 | 1.6 | 0.033 |
| 6319 | SCD | stearoyl-CoA desaturase (delta-9-desaturase) | 1.6 | 0.025 |
| 23657 | SLC7A11 | solute carrier family 7 (anionic amino acid transporter light chain, xc- system), member 11 | 1.6 | 0.004 |
| 85414 | SLC45A3 | solute carrier family 45, member 3 | 1.6 | 0.019 |
| 440905 | LOC440905 | hypothetical LOC440905 | 1.6 | 0.007 |
| 3081 | HGD | homogentisate 1,2-dioxygenase | 1.6 | 0.042 |
| 2537 | IFI6 | interferon, alpha-inducible protein 6 | 1.6 | 0.010 |
| 148823 | C1orf150 | chromosome 1 open reading frame 150 | 1.6 | 0.018 |
| 57126 | CD177 | CD177 molecule | 1.6 | 0.026 |
| 80157 | CWH43 | cell wall biogenesis 43 C-terminal homolog (S. cerevisiae) | 1.6 | 0.030 |
| 957 | ENTPD5 | ectonucleoside triphosphate diphosphohydrolase 5 | 1.7 | 0.004 |
| 10417 | SPON2 | spondin 2, extracellular matrix protein | 1.7 | 0.013 |
| 79054 | TRPM8 | transient receptor potential cation channel, subfamily M, member 8 | 1.8 | 0.022 |
| 11012 | KLK11 | kallikrein-related peptidase 11 | 1.8 | 0.007 |
| 6019 | RLN2 | relaxin 2 | 1.8 | 0.017 |
| 283651 | HMGN2P46 | high mobility group nucleosomal binding domain 2 pseudogene 46 | 1.8 | 0.023 |
| 2346 | FOLH1 | folate hydrolase (prostate-specific membrane antigen) 1 | 1.8 | 0.027 |
| 100506979 | NA | NA | 1.9 | 0.030 |
| 3158 | HMGCS2 | 3-hydroxy-3-methylglutaryl-CoA synthase 2 (mitochondrial) | 2.0 | 0.017 |
| 84419 | C15orf48 | chromosome 15 open reading frame 48 | 2.0 | 0.014 |
| Selection of genes which are up-regulated or down-regulated in the selenium group. Only genes with a fold change <-1.5 or >1.5 are presented. The reported mean fold change and p-value represent expression changes within the selenium group and were calculated using the two-tailed paired Limma t test (p<0.05). Furthermore, all of the presented genes were differentially changed between the selenium and placebo group (one-way ANOVA Limma p<0.05). | | | | |
|  | | | | |
| **PLACEBO** | | | | |
| Entrez ID | Gene symbol | Gene name | Fold change | p-value |
| 6476 | SI | sucrase-isomaltase (alpha-glucosidase) | -2.9 | 0.000 |
| 23671 | TMEFF2 | transmembrane protein with EGF-like and two follistatin-like domains 2 | -2.6 | 0.004 |
| 6013 | RLN1 | relaxin 1 | -2.6 | 0.045 |
| 440905 | LOC440905 | hypothetical LOC440905 | -2.5 | 0.000 |
| 542767 | C1QTNF9B-AS1 | C1QTNF9B antisense RNA 1 (non-protein coding) | -2.4 | 0.001 |
| 119694 | OR51F2 | olfactory receptor, family 51, subfamily F, member 2 | -2.3 | 0.004 |
| 4224 | MEP1A | meprin A, alpha (PABA peptide hydrolase) | -2.2 | 0.013 |
| 7033 | TFF3 | trefoil factor 3 (intestinal) | -2.1 | 0.003 |
| 1807 | DPYS | dihydropyrimidinase | -2.0 | 0.000 |
| 6983 | TRGV9 | T cell receptor gamma variable 9 | -2.0 | 0.015 |
| 56667 | MUC13 | mucin 13, cell surface associated | -2.0 | 0.050 |
| 341883 | LRRC9 | leucine rich repeat containing 9 | -1.9 | 0.004 |
| 6019 | RLN2 | relaxin 2 | -1.9 | 0.010 |
| 54474 | KRT20 | keratin 20 | -1.9 | 0.033 |
| 50940 | PDE11A | phosphodiesterase 11A | -1.8 | 0.003 |
| 54860 | MS4A12 | membrane-spanning 4-domains, subfamily A, member 12 | -1.8 | 0.032 |
| 2044 | EPHA5 | EPH receptor A5 | -1.8 | 0.001 |
| 23310 | NCAPD3 | non-SMC condensin II complex, subunit D3 | -1.8 | 0.032 |
| 338596 | ST8SIA6 | ST8 alpha-N-acetyl-neuraminide alpha-2,8-sialyltransferase 6 | -1.8 | 0.009 |
| 157310 | PEBP4 | phosphatidylethanolamine-binding protein 4 | -1.8 | 0.019 |
| 54898 | ELOVL2 | ELOVL fatty acid elongase 2 | -1.8 | 0.008 |
| 6581 | SLC22A3 | solute carrier family 22 (extraneuronal monoamine transporter), member 3 | -1.7 | 0.008 |
| 33 | ACADL | acyl-CoA dehydrogenase, long chain | -1.7 | 0.003 |
| 1135 | CHRNA2 | cholinergic receptor, nicotinic, alpha 2 (neuronal) | -1.7 | 0.032 |
| 3899 | AFF3 | AF4/FMR2 family, member 3 | -1.7 | 0.006 |
| 4583 | MUC2 | mucin 2, oligomeric mucus/gel-forming | -1.7 | 0.039 |
| 55504 | TNFRSF19 | tumor necrosis factor receptor superfamily, member 19 | -1.7 | 0.002 |
| 4045 | LSAMP | limbic system-associated membrane protein | -1.7 | 0.009 |
| 2980 | GUCA2A | guanylate cyclase activator 2A (guanylin) | -1.7 | 0.005 |
| 11148 | HHLA2 | HERV-H LTR-associating 2 | -1.7 | 0.013 |
| 2168 | FABP1 | fatty acid binding protein 1, liver | -1.6 | 0.004 |
| 10223 | GPA33 | glycoprotein A33 (transmembrane) | -1.6 | 0.016 |
| 57554 | LRRC7 | leucine rich repeat containing 7 | -1.6 | 0.027 |
| 1138 | CHRNA5 | cholinergic receptor, nicotinic, alpha 5 | -1.6 | 0.012 |
| 9576 | SPAG6 | sperm associated antigen 6 | -1.6 | 0.005 |
| 154091 | SLC2A12 | solute carrier family 2 (facilitated glucose transporter), member 12 | -1.6 | 0.001 |
| 353322 | ANKRD37 | ankyrin repeat domain 37 | -1.6 | 0.001 |
| 4285 | MIPEP | mitochondrial intermediate peptidase | -1.6 | 0.002 |
| 5558 | PRIM2 | primase, DNA, polypeptide 2 (58kDa) | -1.6 | 0.018 |
| 29091 | STXBP6 | syntaxin binding protein 6 (amisyn) | -1.6 | 0.005 |
| 6695 | SPOCK1 | sparc/osteonectin, cwcv and kazal-like domains proteoglycan (testican) 1 | -1.6 | 0.006 |
| 81796 | SLCO5A1 | solute carrier organic anion transporter family, member 5A1 | -1.6 | 0.010 |
| 9687 | GREB1 | growth regulation by estrogen in breast cancer 1 | -1.6 | 0.006 |
| 90288 | C3orf25 | chromosome 3 open reading frame 25 | -1.6 | 0.008 |
| 10417 | SPON2 | spondin 2, extracellular matrix protein | -1.6 | 0.047 |
| 1015 | CDH17 | cadherin 17, LI cadherin (liver-intestine) | -1.6 | 0.039 |
| 220965 | FAM13C | family with sequence similarity 13, member C | -1.6 | 0.012 |
| 375611 | SLC26A5 | solute carrier family 26, member 5 (prestin) | -1.6 | 0.000 |
| 22843 | PPM1E | protein phosphatase, Mg2+/Mn2+ dependent, 1E | -1.6 | 0.011 |
| 1573 | CYP2J2 | cytochrome P450, family 2, subfamily J, polypeptide 2 | -1.6 | 0.003 |
| 1767 | DNAH5 | dynein, axonemal, heavy chain 5 | -1.6 | 0.015 |
| 57185 | NIPAL3 | NIPA-like domain containing 3 | -1.5 | 0.049 |
| 79986 | ZNF702P | zinc finger protein 702, pseudogene | -1.5 | 0.024 |
| 1047 | CLGN | calmegin | -1.5 | 0.038 |
| 4133 | MAP2 | microtubule-associated protein 2 | -1.5 | 0.027 |
| 25859 | PART1 | prostate androgen-regulated transcript 1 (non-protein coding) | -1.5 | 0.008 |
| 2651 | GCNT2 | glucosaminyl (N-acetyl) transferase 2, I-branching enzyme (I blood group) | -1.5 | 0.028 |
| 84125 | LRRIQ1 | leucine-rich repeats and IQ motif containing 1 | -1.5 | 0.002 |
| 125981 | ACER1 | alkaline ceramidase 1 | -1.5 | 0.013 |
| 1491 | CTH | cystathionase (cystathionine gamma-lyase) | -1.5 | 0.000 |
| 79846 | C7orf63 | chromosome 7 open reading frame 63 | -1.5 | 0.010 |
| 10846 | PDE10A | phosphodiesterase 10A | -1.5 | 0.020 |
| 29968 | PSAT1 | phosphoserine aminotransferase 1 | -1.5 | 0.012 |
| 39 | ACAT2 | acetyl-CoA acetyltransferase 2 | -1.5 | 0.013 |
| 57715 | SEMA4G | sema domain, immunoglobulin domain (Ig), transmembrane domain (TM) and short cytoplasmic domain, (semaphorin) 4G | -1.5 | 0.011 |
| 81033 | KCNH6 | potassium voltage-gated channel, subfamily H (eag-related), member 6 | -1.5 | 0.013 |
| 11123 | RCAN3 | RCAN family member 3 | -1.5 | 0.016 |
| 11001 | SLC27A2 | solute carrier family 27 (fatty acid transporter), member 2 | -1.5 | 0.045 |
| 100507025 | LOC100507025 | hypothetical LOC100507025 | -1.5 | 0.020 |
| 64757 | MOSC1 | MOCO sulphurase C-terminal domain containing 1 | -1.5 | 0.005 |
| 2786 | GNG4 | guanine nucleotide binding protein (G protein), gamma 4 | -1.5 | 0.038 |
| 23566 | LPAR3 | lysophosphatidic acid receptor 3 | -1.5 | 0.040 |
| 84084 | RAB6C | RAB6C, member RAS oncogene family | -1.5 | 0.028 |
| 79748 | LMAN1L | lectin, mannose-binding, 1 like | -1.5 | 0.017 |
| 23245 | ASTN2 | astrotactin 2 | -1.5 | 0.002 |
| 27284 | SULT1B1 | sulfotransferase family, cytosolic, 1B, member 1 | -1.5 | 0.028 |
| 2104 | ESRRG | estrogen-related receptor gamma | -1.5 | 0.006 |
| 89944 | GLB1L2 | galactosidase, beta 1-like 2 | -1.5 | 0.003 |
| 54733 | SLC35F2 | solute carrier family 35, member F2 | -1.5 | 0.022 |
| 116328 | C8orf34 | chromosome 8 open reading frame 34 | -1.5 | 0.000 |
| 3977 | LIFR | leukemia inhibitory factor receptor alpha | -1.5 | 0.006 |
| 10020 | GNE | glucosamine (UDP-N-acetyl)-2-epimerase/N-acetylmannosamine kinase | -1.5 | 0.003 |
| 3638 | INSIG1 | insulin induced gene 1 | -1.5 | 0.010 |
| 55964 | 37865 | septin 3 | -1.5 | 0.034 |
| 57181 | SLC39A10 | solute carrier family 39 (zinc transporter), member 10 | -1.5 | 0.011 |
| 85391 | SNORD14E | small nucleolar RNA, C/D box 14E | -1.5 | 0.005 |
| 56171 | DNAH7 | dynein, axonemal, heavy chain 7 | -1.5 | 0.000 |
| 202151 | RANBP3L | RAN binding protein 3-like | -1.5 | 0.033 |
| 4685 | NCAM2 | neural cell adhesion molecule 2 | -1.5 | 0.011 |
| 728606 | LOC728606 | hypothetical LOC728606 | -1.5 | 0.006 |
| 3781 | KCNN2 | potassium intermediate/small conductance calcium-activated channel, subfamily N, member 2 | -1.5 | 0.009 |
| 9892 | SNAP91 | synaptosomal-associated protein, 91kDa homolog (mouse) | -1.5 | 0.014 |
| 4983 | OPHN1 | oligophrenin 1 | -1.5 | 0.000 |
| 128344 | C1orf88 | chromosome 1 open reading frame 88 | -1.5 | 0.023 |
| 100124538 | SNORA70C | small nucleolar RNA, H/ACA box 70C (retrotransposed) | -1.5 | 0.011 |
| 4837 | NNMT | nicotinamide N-methyltransferase | 1.5 | 0.017 |
| 1470 | CST2 | cystatin SA | 1.5 | 0.004 |
| 4929 | NR4A2 | nuclear receptor subfamily 4, group A, member 2 | 1.5 | 0.007 |
| 5920 | RARRES3 | retinoic acid receptor responder (tazarotene induced) 3 | 1.5 | 0.006 |
| 2213 | FCGR2B | Fc fragment of IgG, low affinity IIb, receptor (CD32) | 1.5 | 0.002 |
| 714 | C1QC | complement component 1, q subcomponent, C chain | 1.5 | 0.000 |
| 10261 | IGSF6 | immunoglobulin superfamily, member 6 | 1.5 | 0.003 |
| 5328 | PLAU | plasminogen activator, urokinase | 1.5 | 0.001 |
| 4233 | MET | met proto-oncogene (hepatocyte growth factor receptor) | 1.5 | 0.030 |
| 1441 | CSF3R | colony stimulating factor 3 receptor (granulocyte) | 1.5 | 0.002 |
| 3399 | ID3 | inhibitor of DNA binding 3, dominant negative helix-loop-helix protein | 1.5 | 0.003 |
| 25903 | OLFML2B | olfactomedin-like 2B | 1.5 | 0.001 |
| 4064 | CD180 | CD180 molecule | 1.5 | 0.006 |
| 3880 | KRT19 | keratin 19 | 1.5 | 0.005 |
| 27286 | SRPX2 | sushi-repeat containing protein, X-linked 2 | 1.5 | 0.018 |
| 8875 | VNN2 | vanin 2 | 1.5 | 0.026 |
| 5396 | PRRX1 | paired related homeobox 1 | 1.5 | 0.009 |
| 9120 | SLC16A6 | solute carrier family 16, member 6 (monocarboxylic acid transporter 7) | 1.5 | 0.013 |
| 3119 | HLA-DQB1 | major histocompatibility complex, class II, DQ beta 1 | 1.5 | 0.003 |
| 1075 | CTSC | cathepsin C | 1.5 | 0.001 |
| 79750 | ZNF385D | zinc finger protein 385D | 1.5 | 0.034 |
| 963 | CD53 | CD53 molecule | 1.5 | 0.003 |
| 56938 | ARNTL2 | aryl hydrocarbon receptor nuclear translocator-like 2 | 1.5 | 0.006 |
| 8870 | IER3 | immediate early response 3 | 1.5 | 0.000 |
| 58475 | MS4A7 | membrane-spanning 4-domains, subfamily A, member 7 | 1.5 | 0.001 |
| 7474 | WNT5A | wingless-type MMTV integration site family, member 5A | 1.5 | 0.003 |
| 633 | BGN | biglycan | 1.5 | 0.005 |
| 2335 | FN1 | fibronectin 1 | 1.5 | 0.015 |
| 3772 | KCNJ15 | potassium inwardly-rectifying channel, subfamily J, member 15 | 1.5 | 0.004 |
| 3687 | ITGAX | integrin, alpha X (complement component 3 receptor 4 subunit) | 1.5 | 0.003 |
| 80896 | NPL | N-acetylneuraminate pyruvate lyase (dihydrodipicolinate synthase) | 1.5 | 0.001 |
| 27242 | TNFRSF21 | tumor necrosis factor receptor superfamily, member 21 | 1.5 | 0.001 |
| 54209 | TREM2 | triggering receptor expressed on myeloid cells 2 | 1.5 | 0.004 |
| 9332 | CD163 | CD163 molecule | 1.5 | 0.011 |
| 64231 | MS4A6A | membrane-spanning 4-domains, subfamily A, member 6A | 1.5 | 0.000 |
| 1043 | CD52 | CD52 molecule | 1.5 | 0.004 |
| 56925 | LXN | latexin | 1.5 | 0.002 |
| 196 | AHR | aryl hydrocarbon receptor | 1.5 | 0.001 |
| 1012 | CDH13 | cadherin 13, H-cadherin (heart) | 1.5 | 0.016 |
| 1999 | ELF3 | E74-like factor 3 (ets domain transcription factor, epithelial-specific ) | 1.5 | 0.008 |
| 5270 | SERPINE2 | serpin peptidase inhibitor, clade E (nexin, plasminogen activator inhibitor type 1), member 2 | 1.5 | 0.004 |
| 2524 | FUT2 | fucosyltransferase 2 (secretor status included) | 1.6 | 0.022 |
| 55107 | ANO1 | anoctamin 1, calcium activated chloride channel | 1.6 | 0.005 |
| 3397 | ID1 | inhibitor of DNA binding 1, dominant negative helix-loop-helix protein | 1.6 | 0.001 |
| 3394 | IRF8 | interferon regulatory factor 8 | 1.6 | 0.001 |
| 7434 | VIPR2 | vasoactive intestinal peptide receptor 2 | 1.6 | 0.011 |
| 7482 | WNT2B | wingless-type MMTV integration site family, member 2B | 1.6 | 0.026 |
| 1536 | CYBB | cytochrome b-245, beta polypeptide | 1.6 | 0.002 |
| 6337 | SCNN1A | sodium channel, nonvoltage-gated 1 alpha | 1.6 | 0.002 |
| 3689 | ITGB2 | integrin, beta 2 (complement component 3 receptor 3 and 4 subunit) | 1.6 | 0.000 |
| 11006 | LILRB4 | leukocyte immunoglobulin-like receptor, subfamily B (with TM and ITIM domains), member 4 | 1.6 | 0.001 |
| 1959 | EGR2 | early growth response 2 | 1.6 | 0.000 |
| 3726 | JUNB | jun B proto-oncogene | 1.6 | 0.000 |
| 10154 | PLXNC1 | plexin C1 | 1.6 | 0.000 |
| 3934 | LCN2 | lipocalin 2 | 1.6 | 0.019 |
| 9052 | GPRC5A | G protein-coupled receptor, family C, group 5, member A | 1.6 | 0.009 |
| 2207 | FCER1G | Fc fragment of IgE, high affinity I, receptor for /// gamma polypeptide | 1.6 | 0.003 |
| 51338 | MS4A4A | membrane-spanning 4-domains, subfamily A, member 4 | 1.6 | 0.001 |
| 388325 | C17orf87 | chromosome 17 open reading frame 87 | 1.6 | 0.001 |
| 11010 | GLIPR1 | GLI pathogenesis-related 1 | 1.6 | 0.001 |
| 8832 | CD84 | CD84 molecule | 1.6 | 0.001 |
| 3694 | ITGB6 | integrin, beta 6 | 1.6 | 0.001 |
| 1462 | VCAN | versican | 1.6 | 0.027 |
| 6366 | CCL21 | chemokine (C-C motif) ligand 21 | 1.6 | 0.023 |
| 6423 | SFRP2 | secreted frizzled-related protein 2 | 1.6 | 0.001 |
| 1846 | DUSP4 | dual specificity phosphatase 4 | 1.6 | 0.005 |
| 7127 | TNFAIP2 | tumor necrosis factor, alpha-induced protein 2 | 1.6 | 0.005 |
| 4688 | NCF2 | neutrophil cytosolic factor 2 | 1.7 | 0.002 |
| 11326 | VSIG4 | V-set and immunoglobulin domain containing 4 | 1.7 | 0.006 |
| 241 | ALOX5AP | arachidonate 5-lipoxygenase-activating protein | 1.7 | 0.004 |
| 3075 | CFH | complement factor H | 1.7 | 0.002 |
| 121506 | ERP27 | endoplasmic reticulum protein 27 | 1.7 | 0.007 |
| 7805 | LAPTM5 | lysosomal protein transmembrane 5 | 1.7 | 0.000 |
| 4481 | MSR1 | macrophage scavenger receptor 1 | 1.7 | 0.006 |
| 1958 | EGR1 | early growth response 1 | 1.7 | 0.001 |
| 9547 | CXCL14 | chemokine (C-X-C motif) ligand 14 | 1.7 | 0.004 |
| 1520 | CTSS | cathepsin S | 1.7 | 0.000 |
| 6347 | CCL2 | chemokine (C-C motif) ligand 2 | 1.8 | 0.041 |
| 4582 | MUC1 | mucin 1, cell surface associated | 1.8 | 0.004 |
| 7052 | TGM2 | transglutaminase 2 (C polypeptide, protein-glutamine-gamma-glutamyltransferase) | 1.8 | 0.000 |
| 9076 | CLDN1 | claudin 1 | 1.8 | 0.001 |
| 7058 | THBS2 | thrombospondin 2 | 1.8 | 0.039 |
| 5918 | RARRES1 | retinoic acid receptor responder (tazarotene induced) 1 | 1.8 | 0.014 |
| 1118 | CHIT1 | chitinase 1 (chitotriosidase) | 1.8 | 0.005 |
| 28468 | IGHV1-18 | immunoglobulin heavy variable 1-18 | 1.8 | 0.027 |
| 445 | ASS1 | argininosuccinate synthase 1 | 1.8 | 0.000 |
| 6036 | RNASE2 | ribonuclease, RNase A family, 2 (liver, eosinophil-derived neurotoxin) | 1.8 | 0.008 |
| 2191 | FAP | fibroblast activation protein, alpha | 1.8 | 0.020 |
| 51442 | VGLL1 | vestigial like 1 (Drosophila) | 1.8 | 0.002 |
| 2359 | FPR3 | formyl peptide receptor 3 | 1.8 | 0.001 |
| 4023 | LPL | lipoprotein lipase | 1.8 | 0.026 |
| 968 | CD68 | CD68 molecule | 1.8 | 0.000 |
| 5265 | SERPINA1 | serpin peptidase inhibitor, clade A (alpha-1 antiproteinase, antitrypsin), member 1 | 1.8 | 0.007 |
| 7057 | THBS1 | thrombospondin 1 | 1.9 | 0.000 |
| 2212 | FCGR2A | Fc fragment of IgG, low affinity IIa, receptor (CD32) | 1.9 | 0.001 |
| 284340 | CXCL17 | chemokine (C-X-C motif) ligand 17 | 1.9 | 0.003 |
| 6363 | CCL19 | chemokine (C-C motif) ligand 19 | 1.9 | 0.001 |
| 1490 | CTGF | connective tissue growth factor | 1.9 | 0.001 |
| 10457 | GPNMB | glycoprotein (transmembrane) nmb | 1.9 | 0.000 |
| 346389 | MACC1 | metastasis associated in colon cancer 1 | 1.9 | 0.002 |
| 1382 | CRABP2 | cellular retinoic acid binding protein 2 | 1.9 | 0.000 |
| 3855 | KRT7 | keratin 7 | 2.0 | 0.029 |
| 3491 | CYR61 | cysteine-rich, angiogenic inducer, 61 | 2.0 | 0.001 |
| 1311 | COMP | cartilage oligomeric matrix protein | 2.1 | 0.003 |
| 22943 | DKK1 | dickkopf homolog 1 (Xenopus laevis) | 2.1 | 0.000 |
| 6523 | SLC5A1 | solute carrier family 5 (sodium/glucose cotransporter), member 1 | 2.1 | 0.002 |
| 4318 | MMP9 | matrix metallopeptidase 9 (gelatinase B, 92kDa gelatinase, 92kDa type IV collagenase) | 2.2 | 0.007 |
| 5743 | PTGS2 | prostaglandin-endoperoxide synthase 2 (prostaglandin G/H synthase and cyclooxygenase) | 2.2 | 0.003 |
| 2568 | GABRP | gamma-aminobutyric acid (GABA) A receptor, pi | 2.2 | 0.000 |
| 5054 | SERPINE1 | serpin peptidase inhibitor, clade E (nexin, plasminogen activator inhibitor type 1), member 1 | 2.3 | 0.000 |
| 4069 | LYZ | lysozyme | 2.3 | 0.002 |
| 266977 | GPR110 | G protein-coupled receptor 110 | 2.4 | 0.001 |
| 1356 | CP | ceruloplasmin (ferroxidase) | 2.4 | 0.023 |
| 4316 | MMP7 | matrix metallopeptidase 7 (matrilysin, uterine) | 2.5 | 0.039 |
| 6590 | SLPI | secretory leukocyte peptidase inhibitor | 3.0 | 0.000 |
| 1116 | CHI3L1 | chitinase 3-like 1 (cartilage glycoprotein-39) | 3.1 | 0.003 |
| Selection of genes which are up-regulated or down-regulated in the placebo group. Only genes with a fold change <-1.5 or >1.5 are presented. The reported mean fold change and p-value represent expression changes within the placebo group and were calculated using the two-tailed paired Limma t test (p<0.05). Furthermore, all of the presented genes were differentially changed between the selenium and placebo group (one-way ANOVA Limma p<0.05). | | | | |
